# Modulations of Depth Responses in the Human Brain by Object Context: Does Biological Relevance Matter?

**DOI:** 10.1523/ENEURO.0039-21.2021

**Published:** 2021-07-15

**Authors:** Idy W. Y. Chou, Hiroshi Ban, Dorita H. F. Chang

**Affiliations:** 1 Department of Psychology, The University of Hong Kong, Hong Kong, China; 2 Center for Information and Neural Networks (CiNet), National Institute of Information and Communications Technology, Osaka, 565–0871, Japan; 3 Graduate School of Frontier Biosciences, Osaka University, Osaka, 565–0871, Japan; 4 State Key Laboratory of Brain and Cognitive Sciences, The University of Hong Kong, Hong Kong, China

**Keywords:** depth perception, fMRI, object recognition

## Abstract

Depth sensitivity has been shown to be modulated by object context (plausibility). It is possible that it is behavioral relevance rather than object plausibility per se which drives this effect. Here, we manipulated the biological relevance of objects (face or a non-face) and tested whether object relevance affects behavioral sensitivity and neural responses to depth-position. In a first experiment, we presented human observers with disparity-defined faces and non-faces, and observers were asked to judge the depth position of the target under signal-noise and clear (fine) task conditions. In the second experiment, we concurrently measured behavioral and fMRI responses to depth. We found that behavioral performance varied across stimulus conditions such that they were significantly worse for the upright face than the inverted face and the random shape in the signal-to-noise (SNR) task, but worse for the random shape than the upright face in the feature task. Pattern analysis of fMRI responses revealed that activity of fusiform face area (FFA) was distinctly different during depth judgments of the upright face versus the other two stimuli, with its responses (and to a stronger extent, those of V3) appearing functionally-relevant to behavioral performance. We speculate that FFA is not only involved in object analysis, but exerts considerable influence on stereoscopic mechanisms as early as in V3 based on a broader appreciation of the stimulus’ behavioral relevance.

## Significance Statement

We asked how disparity sensitivity is modulated by object (biological) relevance using behavioral and neuroimaging paradigms. We show that behavioral sensitivity to depth-position changes in biological (face) versus non-biological (random surface) contexts, and that these changes are task-dependent. Imaging results highlight a potentially key role of the fusiform region for governing the modulation of stereo encoding by object relevance. These findings highlight powerful interactions between object recognition mechanisms and stereoencoding, such that the utility of disparity information may be up/down weighed depending on the biological relevance of the object.

## Introduction

Binocular disparity, referring to the difference in the left- and right-eye’s image that is a consequence of the two eyes’ separation on the head, is crucial to our everyday ability to recognize and interact with objects (Burke, 2005; Dal Mutto et al., 2011). Neurophysiological work has indicated a wide network of regions in the primate brain, starting from V1, and extending dorsally to V3 and the MT complex, as well as ventrally to V4 and IT, that respond selectively to binocular disparity (Hubel and Wiesel, 1970; Poggio and Fischer, 1977; Poggio and Talbot, 1981; Poggio et al., 1988; Roy et al., 1992; Cumming and Parker, 1997, 1999; DeAngelis et al., 1998; Uka et al., 2000; Hinkle and Connor, 2001). Many of these same areas are responsible for more complex representations rendered by stereoscopic cues. For instance, the caudal intraparietal (CIP) region along dorsal cortex, and V4 and along ventral cortex, respond to 3D slant (Janssen et al., 1999, 2000; Hegdé and Van Essen, 2005; Sakata et al., 2005; Durand et al., 2007).

More recent studies suggest that the particular region engaged during the perception of depth from disparity depends on the task employed. In the primate, the MT complex appears to respond during judgments of disparity signals from noise (DeAngelis et al., 1998; Uka and DeAngelis, 2003, 2004, 2006). On the other hand, V4 and IT seem to be central to the discrimination of fine, but clear depth differences (Uka et al., 2005; Shiozaki et al., 2012). This apparent segregation of the roles of dorsal and ventral regions in different depth discrimination tasks has been observed in humans as well (Chang et al., 2014).

Task-based modulation of stereo responses observed in the cortex suggests that the disparity-processing circuitry is sensitive to context. Recent work has shown that in addition to task requirements, disparity mechanisms can be sensitive to an entirely different form of context, object identity/class. In particular, depth sensitivity is surprisingly modulated by object plausibility (Wong et al., 2020). Specifically, during depth position-judgment tasks, performance was worse for physically plausible versus implausible objects. This effect is particularly striking as both classes of stimuli were equivalently complex, and perhaps more importantly, the plausibility of the object was task-irrelevant. Wong et al. (2020) demonstrated further that the physical plausibility of the object appeared to modulate disparity-related multivariate response patterns in intermediate and higher dorsal (V3A, V3B, V7) and ventral (lateral occipital; LO) regions. These findings highlight the importance of object-level information in processing stereoscopic signals.

Here, we sought to better understand the nature of the dependence of depth responses on object context by asking whether the biological relevance of objects can affect behavioral sensitivity and neural responses to stereoscopic depth. We manipulated the relevance of objects by presenting stereoscopic face stimuli (at upright and inverted orientations) and non-face surfaces matched in depth-power (i.e., amplitude). Indeed, object (biological) relevance has been shown to play a role in the perception of many visual attributes. For instance, the perceived color of food items is biased toward the familiar color of the object stored in long-term memory (Hansen et al., 2006; Olkkonen et al., 2008). Moreover, the race category of a face can affect the perceived lightness of the face (Levin and Banaji, 2006; Chang et al., 2018). The human visual system’s apparent sensitivity to faces is of course now well known to be served by dedicated regions for processing these stimuli, including the fusiform face area (FFA; Kanwisher et al., 1997; McCarthy et al., 1997), the superior temporal sulcus (Puce et al., 1995, 1996), and the occipital face area (Gauthier et al., 2000; Pitcher et al., 2007). Interestingly, the illusory percepts of faces are sufficient to elicit activity in these same regions (Liu et al., 2014).

The apparent importance of faces to our visual system suggests that it is an appropriate stimulus context to be used to explore the effect of biological relevance on disparity processing. In two experiments, we investigated how behavioral sensitivity (experiment 1) and fMRI responses (experiment 2) to depth are modulated by biological relevance. We created random-dot stereograms (RDSs) depicting faces (upright and inverted) and depth-matched random shapes. We elected to include an upside-down variant of the face stimulus as it is well established that faces are more poorly perceived when they are presented inverted (Yin, 1969). Turning the face upside-down thus, presents an interesting stimulus condition that allows us to preserve the structural integrity of an upright face, but is yet reduced in terms of its biological relevance.

## Materials and Methods

### Experiment 1 (behavior)

In experiment 1, we measured depth thresholds in two tasks: a signal-in-noise [signal-to-noise ratio (SNR)] task of judging the depth position (near/far) of a central target relative to the surrounding plane when the SNR varied between 0% and 100%, and a feature task that required discriminating which of two consecutively presented clear objects was nearer. We deemed it important to index performance according to both tasks as they have been shown to engage distinct regions in cortex (Chang et al., 2014; Patten and Welchman, 2015).

#### Participants

A total of 60 observers were recruited for this first experiment: 30 participants (aged 19–34 years; mean 24.9 years; 10 males), were tested on the SNR task and a separate group of thirty participants (aged 18–34 years; mean 22.0 years; 12 males) participated in the feature task. All participants were screened for stereo deficits (Titmus test), had normal or corrected-to-normal vision as screened with the Snellen linear acuity chart, and provided written informed consent in line with ethical approval by the institutional Human Research Ethics Committee (HREC).

#### Apparatus

Stimuli were generated using MATLAB (MathWorks) with extensions from the Psychophysics Toolbox (Brainard, 1997; Pelli, 1997) and presented through a mirror stereoscope, in which two eyes viewed the left and right halves of a 24-inch monitor (1920 × 1080 resolution; 60-Hz refresh rate) through four front-surface mirrors. Viewing distance was 65 cm, and a chinrest was used to limit head movements and maintain viewing distance.

#### Stimuli

**Figure 1.**
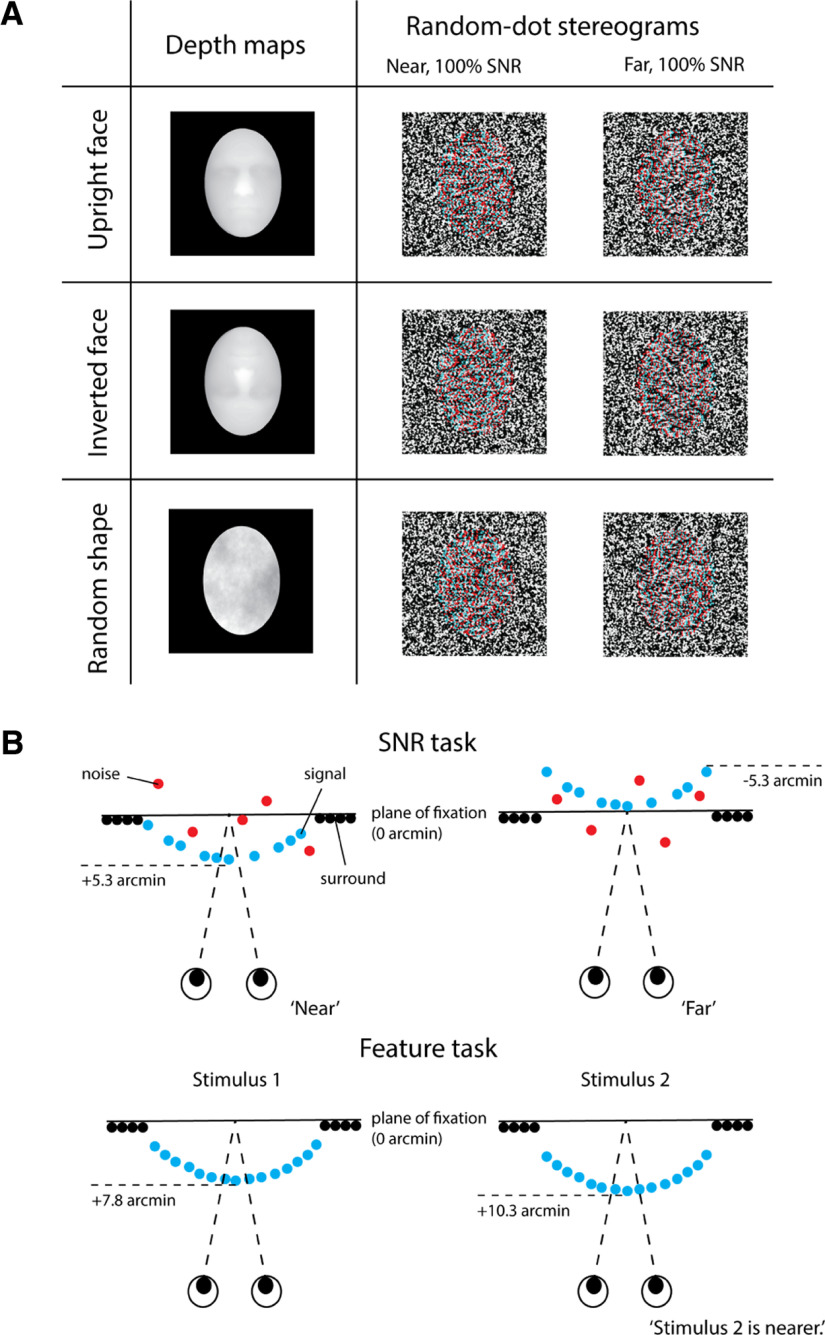
***A***, Sample depth maps and random-dot stereograms (RDS). Top, The depth map of the upright face was presented in an oval region that cropped the ears and the neck. Center, An inverted face was created by rotating the upright face by 180° in the picture plane. Bottom, A depth-power matched random shape was generated by randomizing the phases of the Fourier transform of the upright face while preserving the amplitudes. Anaglyphic examples of the RDS in “near” and “far” conditions, at 100% SNR, are also shown for illustration purposes only. ***B***, In the signal-noise (SNR) task (top), SNR varied between 0% and 100%. In the feature task (bottom), two stimuli were presented consecutively on each trial. Disparity difference varied within 150 arcs.

Three kinds of stimuli, including the upright face, the inverted face, and the depth-power-matched random shape, were rendered as RDSs (Fig. 1*A*). 3D laser-scanned head models were firstly obtained from the Face Database of the Max Planck Institute for Biological Cybernetics (Troje and Bülthoff, 1996; Blanz and Vetter, 1999). All heads were in frontal view with hair removed. Four face identities, including two male and two female head models, were randomly selected and rendered as depth maps such that intensity coded for depth. The size and shape of all faces were standardized by applying an oval-shape cropping mask that eliminated the ears and the neck. The inverted faces were generated by rotating the upright faces by 180° in the picture plane. The depth-power-matched random shapes were created by taking the depth map of each upright face, applying a Fourier transform, and randomizing its phases while preserving the amplitudes. The resulting images thus carry no structural coherence but maintain identical depth information as the upright and inverted variants. A total of four random shapes were created, each corresponding to one face identity.

The depth maps were finally rendered as RDSs, where the amount of pixel-wise disparity was indexed by intensity. Each RDS had a maximum disparity of ±5.3 arcmin and a size of 8.68° × 8.68°, within which the target subtended 6.45° vertically and 4.55° horizontally. Each target had an average disparity of ±4.27 arcmin. Dots were randomly assigned as black or white, with a size of 0.05° and a density of 20 dots/deg^2^. The RDS was presented against a black background and surrounded by a grid of gray and white squares (each 0.5° in size) that served to provide an unambiguous background reference and stabilize vergence position.

#### Tasks

##### SNR task

On each trial, participants were required to judge whether a central target was “near” or “far” as compared with the surrounding region (the “surround”). Disparity of the surround was fixed at zero (i.e., at the plane of fixation). For the target, disparity was fixed at ±5.3 arcmin at the maximum point across the target’s surface. Task difficulty was manipulated by varying the SNR, altering the proportion of dots that defined the target (“signal”) relative to noise dots that were assigned a random disparity within the range of ±5.3 arcmin (Fig. 1*B*). The initial test value was 80% SNR. On each trial subsequently, the SNR of the stimulus was adjusted according to the QUEST staircase procedure, estimating thresholds at the 82% correct level (Watson and Pelli, 1983).

##### Feature task

On each trial, participants were required to judge which of two consecutively presented objects was “nearer”. One of the objects was designated the “reference” and had a fixed disparity of 7.8 arcmin. Task difficulty was manipulated by varying the disparity difference between the target and the reference within a range of 150 arcs (Fig. 1*B*). Both the target and reference had crossed disparity and were presented at 100% SNR. The initial test value of the target was set at 120 arcs (relative to the reference). As for the SNR task, on each trial, disparity difference was adjusted according to the QUEST staircase procedure yielding threshold estimates at the 82% accuracy level (Watson and Pelli, 1983).

Before each task, participants were provided with oral instructions, viewed examples of the stimuli, and completed twelve practice trials with audio feedback to get familiarized with the tasks. Each task consisted of six test runs [three conditions (upright face, inverted face, random-surface) × two stimulus identities] presented in random order. A test run contained one type of object with two interleaved staircases of stimulus gender (i.e., the gender of the head models from which the stimuli were derived). Each staircase had 64 trials that comprised of four practice trials and 60 test trials. The test value of the first test trial was determined by the threshold obtained in the practice trials.

In both tasks, each trial began with a nonius-type fixation box (0.25° in size) that consisted of dichoptically presented nonius lines (each eye perceives only two of the four lines), which lasted for 500 ms. In the SNR task, each stimulus was presented for 300 ms. In the feature task, each stimulus was presented for 300 ms with an interstimulus interval of 500 ms. In both tasks, stimulus presentation was followed by a response period that ended after a response was made or 3000 ms, whichever came first. Trials without a response after the maximum response period elapsed were deemed incorrect. Participants were required to respond using the arrow keys on a computer keyboard.

### Experiment 2 (fMRI)

In experiment 2, we measured fMRI responses when subjects were performing the SNR task, and examined both univariate and multivariate responses to depth under the different stimulus conditions. In this second experiment, we selected to test the SNR task as it elicited the more robust behavioral effects both here, and in previous work (Wong et al., 2020).

#### Participants

Twenty-two participants (aged 21–34; mean 23.2 years; 17 males) who did not participate in the behavior experiments were recruited for the fMRI experiment. All participants had normal or corrected-to-normal vision and provided written informed consent in line with ethical approval by the institutional HREC.

#### Apparatus

Left and right images were back-projected using two projectors (WUX4000, Canon), each equipped with a polarizing filter (resolution: 1280 × 1024 pixels; 60-Hz refreshing rate) and placed at 96 cm from the back of the fMRI bore. Participants viewed the stimuli with the corresponding polarized glasses through a 45° tilted mirror mounted in front of the head.

#### Stimuli

Stimuli were the same as those used in experiment 1 except for the following differences. First, for each condition (upright face/inverted face/random shape), only two stimulus exemplars were used, one per stimulus gender, resulting in a total of six unique images used in this experiment. This change was made to minimize the noise of fMRI signals across stimulus conditions that could result with large stimulus variance. Second, the target subtended 5.23° vertically and 3.70° horizontally to accommodate a smaller field of view in the bore setup. Third, the maximum disparity on the target surface was 5.4 arcmin.

#### fMRI acquisition

Imaging data were acquired at the Center for Information and Neural Networks (CiNet), National Institute of Information and Communications Technology (NICT), Osaka, Japan, using a 3-T Siemens Trio MR scanner with a half of the 32-channel, phase-array (whole) head coil that covered the occipital lobe. Head movements were limited by a foam padding inside the coil. For both localizer and experimental runs, blood oxygenation level-dependent (BOLD) signals were measured with a multiband echoplanar imaging (EPI) sequence [voxel size = 2 × 2 × 2 mm^3^, echo time (TE) = 30 ms, repetition time (TR) = 2000 ms, field of view = 192 × 192, flip angle = 75°, 78 axial slices (slightly oblique along the AC–PC line), multiband factor = 3; 205 volumes where the first 5 were discarded to eliminate the effects of start-up transients] provided by the University of Minnesota (under a C2P contract). Additionally, high-resolution T1-weighted images were collected for each participant (voxel size = 1 mm^3^, TE = 2.48 ms, TR = 1900 ms, field of view = 256 × 256, flip angle = 9°, 208 slices).

#### Region of interest (ROI) definition

For each participant, we defined ROIs V1, V2, V3, V4, V3A, and V7 using standard phase-encoded retinotopic mapping procedures that mapped polar angle with a checkerboard wedge stimulus that was rotating periodically (clockwise or anti-clockwise; https://github.com/hiroshiban/Retinotopy; Sereno et al., 1995). Area V3B/KO (kinetic occipital) was defined using the same map, as the region falling anterior to V3A and inferior to V7 (DuPont et al., 1997). Using a single-run functional localizer, we identified hMT+ (human motion complex) as a set of contiguous voxels that showed significantly stronger responses (*p *<* *0.01) to an array of moving dots that receded or expanded coherently than to an array of static dots (Huk et al., 2002). Note that because of time constraints, five participants did not complete the hMT+ localizer scan. For these participants, we defined hMT+ as fixed 5-mm spherical ROIs centered on Talairach coordinates of [−51, −72, 0] and [51, −69, 3] in the left and right hemispheres, respectively (Orban et al., 2003). LO was identified as the region in the lateral occipito-temporal cortex that was significantly more responsive (*p *<* *0.01) to intact than scrambled images of objects and shapes (Kourtzi and Kanwisher, 2001). Bilateral FFAs were defined as 5-mm spherical ROIs for all participants, with Talaraich coordinates of [−37, −42, −16] in the left hemisphere and [39, −40, −16] in the right hemisphere (Grill-Spector et al., 2004). Note that FFA was not localized in individual subjects as we saw no reason to extract responses from solely face-stimulus-specific voxels in this region.

#### Design and procedures

Before scanning, each participant firstly completed three runs of the SNR task inside the MRI bore (with the scanner idle). Thresholds estimated from these in-bore behavior-only runs were used to determine individually-tailored stimulus test values for subsequent fMRI acquisition runs. This was done to standardize task difficulty across conditions and participants, thus reducing the noise of fMRI signals in our data. From these behavior-only runs, we obtained subject-specific test values for the fMRI runs as follows: for each staircase (stimulus condition), we took the average of the test values in last 30 trials and defined a range of ±1 SD from this mean value. Subsequently, on each fMRI trial (of the corresponding condition), the SNR value was randomly sampled within this range. For any instances where a single staircase could not yield reliable estimates [because of: (1) a lack of asymptotic convergence, and/or (2) 95% confidence intervals reaching floor] stimuli were sampled at 80% SNR in the corresponding fMRI trials. This implicated a total of 19 (of 132 total staircases) run in bore. fMRI runs were arranged in a block design. Each run comprised seven block types, corresponding to six stimulus conditions [3 (type) × 2 (gender)] and fixation. Each block lasted for 16 s. A stimulus block comprised 8 trials with equal number of “near” and “far” trials. On each trial, a stimulus was presented for 500 ms and was followed by a fixed-duration response period of 1500 ms. A background grid of randomly gray and white squares was present throughout the entire run.

A fixation block contained a nonius-type fixation (see above for details). In each run, each stimulus block type was repeated twice, with condition order randomized, and interleaved with fixation blocks. Thus, there were a total of 12 stimulus blocks and 13 fixation blocks (each run began and ended with a fixation block). Each run consisted of 96 trials and lasted 6.4 min. The entire scanning session lasted around 90 min.

#### fMRI data analysis

Data were processed using BrainVoyager QX (Goebel et al., 2006; Formisano et al., 2006). For each participant, T1-weighted anatomic scans were transformed into Talairach space (Talaraich and Tournoux, 1988). Inflated and flattened cortical surfaces were reconstructed for both hemispheres. Functional data were preprocessed with slice time correction, 3D rigid-body motion correction, linear trend removal and high-pass filtering (frequency space filter; three cycles per run) without spatial smoothing.

We performed two analyses that examined both univariate [general linear model (GLM)] responses and pattern-level responses using multivoxel pattern analysis (MVPA). The GLM consisted of three regressors representing the three stimulus conditions (upright face, inverted face and random shape) and six regressors of motion parameters (three translation parameters and three rotation parameters) and a constant term. Stimulus-condition regressors were defined by modeling stimulation periods separately for each stimulus condition by a boxcar model convolved with a (two-γ) hemodynamic response function (HRF; Glover, 1999). The averaged time course signal across all voxels of each ROI was then modelled as a linear combination of the regressors. The regressor coefficients of different stimulus conditions and ROIs were then contrasted.

MVPA classification analysis was done with a linear support vector machine implemented in libSVM tool (Chang and Lin, 2011) with a recursive feature elimination (RFE) technique (De Martino et al., 2008). The RFE is a multivariate feature selection algorithm that eliminates uninformative voxels. The time course signals of all voxels were converted into *z* scores. Also, to account for the typical delay of the hemodynamic response, signals were shifted by 4 s (= 2 TRs; Serences, 2004). For each ROI, the SVM was trained to classify the patterned responses between the upright face and the random shape, between the inverted face and the random shape, and between the upright face and the inverted face.

A leave-one-run-out cross-validation procedure was used for the MVPA. Specifically, in each cross-validation, functional data of one run were used as test dataset and the rest of the data were used as training dataset for computing SVM weights. In each RFE step, the SVM classifier was trained with 80% of the training dataset, which were resampled 20 times without partition of blocks. Therefore, each voxel was assigned with 20 SVM weights. All voxels were ranked according to the average weights in that particular RFE step, and the five voxels that had the lowest average weights were eliminated. The remaining data were used to decode the test pattern, and the prediction accuracy for that particular voxel pattern (in the particular RFE step) was obtained. This procedure was repeated until the voxel count fell below 250, after which it would proceed to the next cross-validation. As such, the final pattern size for all ROIs here was 250 voxels. Note that the analysis was in fact repeated at all possible fine voxel counts between 50 and 800 voxels (stepping in 50 voxel increments), but we ultimately decided to report results from the 250 pattern size as this corresponded to the smallest pattern size at which accuracies reached asymptotic levels. For each ROI and classification, the final prediction accuracy was tested against permutation baseline level (0.53), as determined by running 1000 SVMs with shuffled labels. In addition to an ROI-based MVPA, we conducted a searchlight analysis in which the pattern discriminability between stimulus conditions was tested by moving a 6-mm (radius) spherical ROI across the cortex.

We explored the relationship between MVPA classification accuracies and behavioral thresholds. For each set of comparisons [(1) upright face vs random shape, (2) inverted face vs random shape, and (3) upright face vs inverted face], we extracted its SVM accuracy and calculated a behavioral index, corresponding to the difference in behavioral thresholds between the two conditions in the SNR task. We then computed the (Pearson’s) correlation coefficient (*r*) between the two metrics for each ROI. We also performed a further analysis concatenating the inverted face and random shape conditions, forming a “non-upright-face” condition. The corresponding SVM was run, and accuracies subsequently correlated with a behavioral index similarly computed by averaging thresholds from the two non-upright-face conditions.

## Results

### Behavior

**Figure 2.**
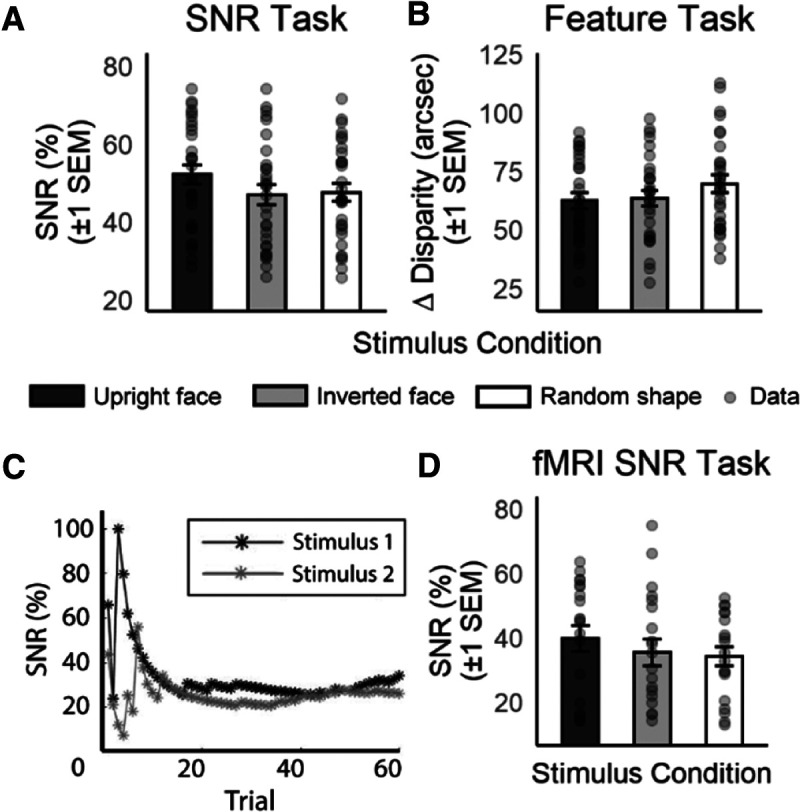
***A***, ***B***, Depth discrimination thresholds in the signal-noise (SNR) and feature tasks. In the SNR task, thresholds were higher (i.e., poorer) for the upright face than the inverted face and the random shape. In the feature task, thresholds for the upright face were significantly lower (i.e., better) than those for the random shape, whereas thresholds for the inverted face did not differ significantly from those of the other two stimulus conditions. ***C***, Sample staircases from a randomly-sampled observer, showing threshold estimates across trials within one experimental run, for the upright face condition. ***D***, Behavioral thresholds obtained in-bore (SNR task). Thresholds were higher for the upright face than the random shape. Individual subject thresholds are overlaid in ***A–C***. Error bars represent ±1 SEM.

Behavioral performance for the SNR and feature tasks, indexed in terms of signal-noise (SNR task) or disparity difference thresholds (feature task) are presented in Figure 2. Sample staircases from one subject are shown in Figure 2*C*. Thresholds for each task were analyzed using a one-way repeated-measures ANOVA comparing thresholds across stimulus conditions (Table 1). The analysis for the SNR task (Fig. 2*A*) indicated a significant main effect of stimulus condition, *F*_(2,58)_ = 5.65, *p *=* *0.006, $\eta_{p}^{2}$ = 0.163. *Post hoc* Bonferroni-corrected *t* tests revealed that thresholds were higher (poorer) for the upright face than the inverted face, *t*_(29)_ = 3.00, *p *=* *0.018, *d *=* *0.547 and for the upright face than the random shape, *t*_(29)_ = 2.89, *p *=* *0.021, *d* = 0.528. However, there was no significant difference between the thresholds for the inverted face and that for the random shape *t*_(29)_ = 0.337, *p *>* *0.999, *d *=* *0.067.

**Table 1:** Statistical table

Analysis	Figure	Data structure	Type of test	Variables	Observed power
Experiment 1, SNR task	2*A*	Assumed normal	One-way repeated- measures ANOVA	Independent: stimulus condition Dependent: depth threshold	0.843
Experiment 1, feature task	2*B*	Assumed normal	One-way repeated- measures ANOVA	Independent: stimulus condition Dependent: depth threshold	0.722
Experiment 2, behavior	2*D*	Assumed normal	One-way repeated- measures ANOVA	Independent: stimulus condition Dependent: depth threshold	0.610
Experiment 2, GLM	3	Assumed normal	Three-way repeated- measures ANOVA	Independent: hemisphere Stimulus condition ROI Dependent: GLM β-weight	0.580
					0.372
					>0.999
Experiment 2, MVPA	4	Assumed normal	One-sample *t* test	SVM accuracy against baseline
				Upright face vs random shape classification
				V1	0.690
				V2	>0.999
				V3	0.972
				V3A	0.744
				V3B	0.422
				V7	0.919
				hMT+	0.703
				V4	0.880
				LO	0.905
				FFA	0.762
				Inverted face vs random shape classification
				V1	0.959
				V2	0.997
				V3	0.639
				V3A	0.999
				V3B	0.479
				V7	0.998
				hMT+	0.175
				V4	0.999
				LO	0.764
				FFA	0.396
				Upright face vs inverted face classification
				V1	0.437
				V2	0.418
				V3	0.064
				V3A	0.999
				V3B	0.875
				V7	0.504
				hMT+	0.088
				V4	0.903
				LO	0.540
				FFA	0.838
Experiment 2, MVPA- behavior correlation	4	Assumed normal	Pearson correlation	Variable 1: Threshold difference
				Variable 2: SVM accuracy
				Upright face vs random shape
				V1	0.050
				V2	0.378
				V3	0.288
				V3A	0.071
				V3B	0.055
				V7	0.074
				hMT+	0.051
				V4	0.690
				LO	0.258
				FFA	0.075
				Inverted face vs random shape
				V1	0.312
				V2	0.087
				V3	0.087
				V3A	0.579
				V3B	0.117
				V7	0.342
				hMT+	0.053
				V4	0.053
				LO	0.075
				FFA	0.072
				Upright face vs inverted face
				V1	0.533
				V2	0.449
				V3	0.984
				V3A	0.058
				V3B	0.353
				V7	0.250
				hMT+	0.202
				V4	0.488
				LO	0.193
				FFA	0.544
				Upright face vs non-upright-face
				V1	0.057
				V2	0.129
				V3	0.170
				V3A	0.060
				V3B	0.110
				V7	0.065
				hMT+	0.061
				V4	0.121
				LO	0.064
				FFA	0.562

The analysis for feature task thresholds (Fig. 2*B*) revealed a significant main effect of stimulus condition, *F*_(2,58)_ = 4.26, *p *=* *0.019, $\eta_{p}^{2}$ = 0.128. *Post hoc* Bonferroni-corrected *t* tests indicated that thresholds were significantly lower (better) for the upright face than the random shape *t*_(29)_ = 2.79, *p *=* *0.027, *d *=* *0.510, but there was no significant difference between the thresholds for the inverted face and the upright face, *t*_(29)_ = 0.431, *p *>* *0.999, *d *=* *0.079, and that between the inverted face and the random shape, *t*_(29)_ = 2.04, *p *=* *0.153, *d *=* *0.372.

### fMRI

#### In-bore behavior (SNR task)

We verified firstly that behavioral effects for the task chosen for the MRI could be replicated by the new group of participants while inside the bore. A one-way repeated-measures ANOVA indicated a significant effect of stimulus condition, *F*_(2,36)_ = 3.45, *p *=* *0.043, $\eta_{p}^{2}$ = 0.161. *Post hoct* tests indicated that, consistent with findings from experiment 1, thresholds for the upright face were significantly higher than those for the random shape: *t*_(18)_ = 2.33, *p *=* *0.032, *d *=* *0.448, but there were no significant differences between thresholds for the upright face and the inverted face (*t*_(18)_ = 1.74, *p *=* *0.098, *d *=* *0.244), and between the inverted face and the random shape (*t*_(18)_ = 1.05, *p *=* *0.309, *d *=* *0.169).

Turning to the threshold-sampled stimuli of the functional runs, we then assessed whether the difference in disparity distribution between stimuli at the “near” and “far” stimulus positions (relative to the fixation plane) might have an influence on response accuracies by means of a 2 (depth-position: near/far) × 3 (condition) ANOVA. The analyses indicated no significant main effect of depth-position, *F*_(1,21)_ = 0.053, *p* = 0.820, $\eta_{p}^{2}$ = 0.003, nor interactions involving depth-position [mean accuracy (SD) for Near_uprface_ = 0.81 (0.13); Far_uprface_ = 0.79 (0.18); Near_invface_ = 0.80 (0.17); Far_invface_ = 0.81 (0.15); Near_ran_ = 0.78 (0.13); Far_ran_ = 0.82 (0.13)].

#### GLM

**Figure 3.**
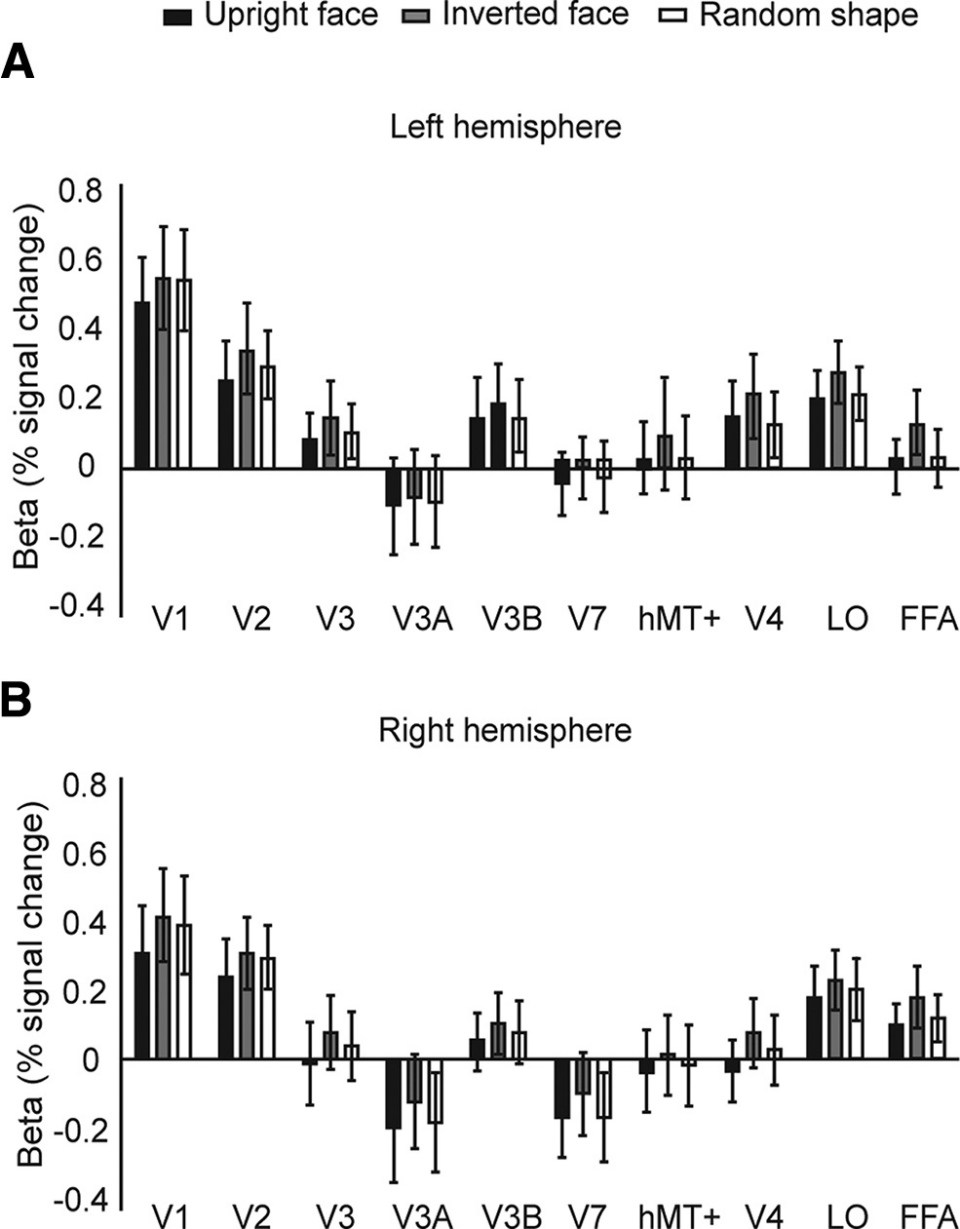
GLM β-weights (% signal change) of the three stimulus conditions in the (***A***) left and (***B***) right hemispheres. Univariate signals were globally higher in the left hemisphere than the right hemisphere. Signals were generally higher in V1, V2, and LO. Error bars represent ±1 SEM (*N* = 22).

We firstly examined univariate fMRI signals across stimulus conditions. For each hemisphere, we extracted the GLM β-weights (percent signal change) corresponding to the three conditions from the ten ROIs (Fig. 3). These were then entered into a 2 (hemisphere) × 3 (type) × 10 (ROI) repeated-measures ANOVA (Table 1) that revealed significant main effects of hemisphere (*F*_(1,21)_ = 5.28, *p *=* *0.032, $\eta_{p}^{2}$ = 0.201) and ROI (*F*_(9,189)_ = 7.60, *p *<* *0.001, $\eta_{p}^{2}$ = 0.266). β-Weights did not differ across conditions (*F*_(2,42)_ = 1.87, *p *=* *0.167, $\eta_{p}^{2}$ = 0.082), and there were no significant interactions: hemisphere × condition: *F*_(2,42)_ = 0.609, *p *=* *0.549, $\eta_{p}^{2}$ = 0.028; hemisphere × ROI: *F*_(9,189)_ = 0.656, *p *=* *0.748, $\eta_{p}^{2}$ = 0.030; ROI × condition: *F*_(18,378)_ = 0.610, *p *=* *0.892, $\eta_{p}^{2}$ = 0.028; hemisphere × condition × ROI: *F*_(18,378)_ = 0.1.37, *p *=* *0.141, $\eta_{p}^{2}$ = 0.036. A comparison of the mean β-weights indicated that signals were generally stronger in the left hemisphere (M* *=* *0.145, SE* *=* *0.083) than the right hemisphere (M* *=* *0.073, SE* *=* *0.079).

Note that while the GLM results indicated stronger responses in the left hemisphere as compared with the right hemisphere, amplitudes differences were homogenous across ROIs and stimulus conditions. For this reason, and, as subsequent multivariate analyses remove the univariate amplitude, we elected to concatenate data from the two hemispheres for subsequent analyses.

#### MVPA

**Figure 4.**
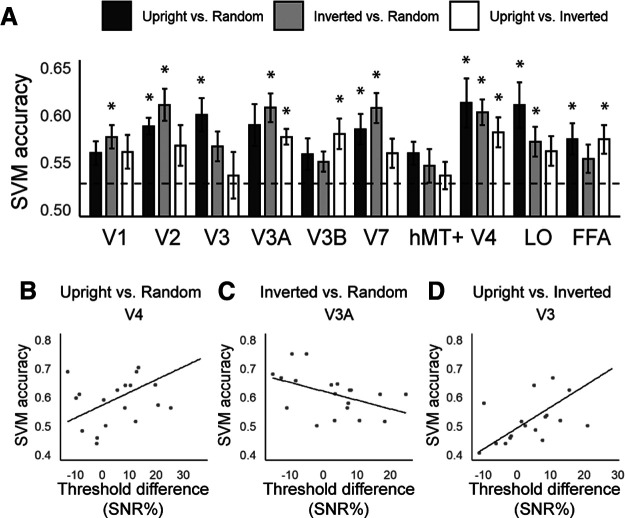
***A***, SVM accuracies for all ROIs in the three exhaustive classifications comparing all stimulus conditions. Classification accuracies (Bonferroni-corrected) that are significantly above-baseline (0.527; dashed line) are indicated by asterisks. Error bars represent ±1 SEM (*N* = 22). ***B–D***, Brain-behavior correlations in V4 for upright-face versus random-shape (***B***); V3A for inverted-face versus random-shape (***C***); and V3 for upright-face versus inverted-face (***D***). The horizontal axis represents a behavioral index computed as the threshold difference between conditions. Additional results from the whole-brain classification (searchlight) and from an additional brain-behavior correlation following the concatenation of the inverted and non-face conditions (FFA) are presented in Extended Data Figures 4-1, 4-2, respectively.

Multivariate responses to different stimulus conditions were examined by contrasting the multivoxel fMRI signals between the upright face and the random shape, and those for the inverted face and the random shape (see Materials and Methods). MVPA classification accuracies are presented in Figure 4*A*. Classification accuracies were compared with baseline (0.53) while holding familywise error rate at 0.05 (qFDR < 0.05; Table 1).

10.1523/ENEURO.0039-21.2021.f4-1 Extended Data Figure 4-1 Sample searchlight maps for SVM discriminations between the upright face and random shape conditions. Results are superimposed onto the representative flattened surface maps of a single participant with ROIs delineated. Gyri are colored in light grey and sulci in dark grey. Download Figure 4-1, TIF file.

10.1523/ENEURO.0039-21.2021.f4-2 Extended Data Figure 4-2 Brain-behavior correlation in FFA for the upright-face versus non-face (i.e., concatenating the inverted face and random shape conditions) comparison. The horizontal axis represents a behavioral index computed as the threshold difference between the upright face condition and the mean of the two non-face conditions. Download Figure 4-2, TIF file.

SVM accuracies for the classification between the upright face and the random shape were significantly above permutation-baseline in V2, V3, V7, V4, LO, and FFA, while those between the inverted face and the random shape were above-baseline in V1, V2, V3A, V7, V4, and LO. For the comparison between the upright face and inverted face, SVM accuracies were above-baseline in V3A, V3B, V4, and FFA. In other words, brain regions that had above-baseline classification performance in the contrasts involving the random-shape (which may reflect simply the classification of differences in lower-order features) included V2, V7, V4, and LO. Regions that showed above-baseline discriminability for the contrast between the upright face and the random shape only were V3 and FFA, while those above-baseline for the contrast between the inverted face and the random shape only were V1 and V3A. Only V3B uniquely distinguished between responses of the upright versus inverted faces. Finally, only FFA exhibited above-baseline discrimination accuracies that were common and unique to both SVMs involving the upright face stimulus (i.e., above-baseline for both the upright face vs random, and upright face vs inverted face comparisons, but not the inverted vs random comparison).

Lastly, the searchlight analysis reflected that our choice of ROIs well covered the regions showing above-baseline pattern discriminability. Sample searchlight maps for the pattern discriminability (*t* value) between the upright face and random shape conditions are presented in Extended Data Figure 4-1. Note that these searchlight maps were not further used to emphasize our main findings together with the other results to avoid circularity problems (Kriegeskorte et al., 2009).

#### Brain-behavior relationships

Finally, to clarify the relevance of these brain areas for the effects observed behaviorally, we further examined the relationship between behavioral thresholds and MVPA accuracies within each ROI (Fig. 4) in two sets of analyses. First, for each possible comparison of the three stimulus conditions [(1) upright face vs random shape, (2) inverted face vs random shape, (3) upright face vs inverted face], we derived a behavioral index computed as the difference of SNR thresholds between the two conditions. We then calculated the (Pearson’s) correlation coefficient (*r*) between this behavioral index and the SVM accuracies from the classification of the corresponding stimulus conditions for each ROI across all participants. We found that for the “upright face versus random shape” comparison, SVM accuracies in V4 correlated positively to the behavioral index (*r *=* *0.513, *p *=* *0.025); for the “inverted face versus random shape” comparison, SVM accuracies in V3A correlated negatively to behavioral index (*r* = –0.465, *p *=* *0.045); and finally, for the “upright face versus inverted face” comparison, SVM accuracies in V3 correlated positively to the behavioral index (*r *=* *0.707, *p *<* *0.001). Amongst these results, only the last involving V3 withstood Bonferroni correction for multiple comparisons.

Second, we performed an additional MVPA-behavior correlational analysis with data of the inverted face and random shape conditions combined to form a “non-upright-face” condition. Indeed, previous work has suggested that upright faces represent a class of objects that is distinct from non-face objects, as well as inverted faces (Yin, 1969; Valentine, 1988; Farah et al., 1995; Kanwisher et al., 1997; Kanwisher and Yovel, 2006). Behavioral indices (upright face minus non-upright-face conditions) were then correlated with results from a separate MVPA that compared classification accuracies for discriminating between the upright face and non-upright-face (i.e., inverted face + random shape) conditions. We found a significant correlation between behavior and SVM accuracies in FFA only (*r *=* *0.481, *p *=* *0.041; Extended Data Fig. 4-2), although the correlation did not survive further statistical correction.

## Discussion

We tested for differences in behavioral sensitivity and fMRI responses to stereoscopic depth, in face and non-face contexts. In experiment 1, we compared behavioral depth discrimination performances under three conditions with varying biological relevance: upright faces, inverted faces, and phase-randomized surfaces. We showed that the behavioral sensitivity to depth differed across conditions, but curiously, also across tasks. In the SNR task, thresholds were higher for the upright face than the inverted face and the random shape; that is, SNR depth sensitivity was poorer in a biologically relevant context. In contrast, in the feature task, depth thresholds were higher for the random shape than the upright face; that is, fine thresholds were instead, better for biologically relevant stimuli. In experiment 2, we selected the SNR task, as it elicited more robust differences in responses across stimulus contexts, both in experiment 1 of the present study and in previous work (Wong et al., 2020), and examined fMRI responses to the stimuli concurrently with behavior. Analysis of the univariate responses showed rather homogenous activation across stimulus conditions. However, results from the MVPA were more revealing. Early and intermediate retinotopic areas (V1, V2, V3, V3A, V3B, V4) and extrastriate dorsal (V7) and ventral (LO, FFA) regions showed discriminable response patterns across contexts. Notably, of these regions, responses of FFA in particular appear to be classifying information beyond simple lower-order features (i.e., are uniquely and commonly discriminative for contrasts that involved the upright face). Moreover, multivariate responses of V3, V4 and FFA are predictive (albeit weakly) of behavioral responses. We consider the behavioral and imaging findings in turn.

### Modulation of behavioral sensitivity to depth by object relevance

We firstly consider our behavioral findings. Our data indicate that depth sensitivity differs between face and non-face contexts and, importantly, this difference is task-dependent. Our results showed that a face context, in particular in the upright orientation, facilitates feature (disparity) discrimination but worsens the segmentation of disparity signal from noise. The reversed trends observed between the tasks, at first glance, may seem paradoxical. However, as it has been proposed that performance in tasks similar to those employed here can be thought to involve two different mechanisms, namely noise filtering and feature readout (Dosher and Lu, 2005), we propose that the differences between the SNR task and feature task can be explained within this framework. The level of external noise is high in the SNR task but absent in the feature task, implying that noise filtering is the dominating visual process during the SNR task but not the feature task. The two tasks also require different levels of precision: the SNR task requires a lower level of precision (near/far depth discrimination), whereas the feature task requires a higher level of precision (discrimination of fine depth difference). This implies a heavy engagement in feature readout for the feature task, but only a modest amount of feature extraction could suffice depth judgments in the SNR task.

Why then, is noise filtering better in non-face objects, and feature readout better in face contexts? First, that the detection of a target from noise is better for novel, unfamiliar objects has been well documented in the literature. For example, when participants are presented with an array of four objects and then asked to recall the location of an item in the array, accuracy is significantly higher when the test item is novel than when it is familiar (Johnston et al., 1990). This “novel popout” effect, referring to the fact that *novel* objects are easier to detect when noise is present, may relate to the higher saliency of novel (vs familiar) objects that captures attention and hence facilitates target detection. In our SNR task, the “novel popout” effect can be manifest as an improvement in the quality of signal extraction for the random shape (compared with the upright face), thus reducing thresholds. Furthermore, it has been shown that the detection of faces appears to be especially susceptible to noise compared with other non-face daily objects (Chen et al., 2015). In particular, the presence of noise impairs feature discrimination to a larger extent for faces than for cars. In the context of this previous work then, our data for the SNR task appear to be in alignment.

Second, we found that feature readout is better for the upright face than for the random shape. In everyday functioning, the readout of fine features is crucial to face individuation and the recognition of facial expressions. Indeed, that a higher level of precision is required for the perception of faces than other objects has been shown empirically. Participants inclined to name faces at the level of its identity (i.e., the name of the face), but dogs at the level of its species or subspecies (e.g., “golden retriever” or simply “dog”), indicating that the processing of faces, compared with other objects, is more specific (Tanaka, 2001). Other work has shown that human participants are strikingly sensitive to fine differences in faces (Lehky, 2000). It is thus very likely that fine feature readout is more relevant to everyday face perception tasks. By contrast, precise feature discrimination is rarely required for non-face objects. Here, it is important to note that any behavioral enhancements of feature readout in face contexts shall not necessarily manifest in the results of the SNR task as only a limited amount of feature readout was needed in this task.

Finally, we observed a clear difference of thresholds between the upright and inverted faces in the SNR task. This is in line with the well-documented face inversion effect, in which the inversion of face affects perception, both in terms of recognition performance (Yin, 1969; Valentine, 1988; Farah et al., 1995) and neural responses (Kanwisher et al., 1998) of face-selective regions. Yet, behavioral thresholds did not differ substantially between the upright face and the inverted face in the feature task. Interestingly, these findings seem to suggest that the face inversion effect is more prominent in high noise, compared with the discrimination of fine feature differences in low visual noise.

Importantly, the opposite behavioral trends observed in our SNR and feature tasks distinguishes these findings from those observed by Wong et al. (2020), who showed that the physical plausibility of objects affects depth sensitivity similarly for SNR and feature discrimination tasks. Particularly, depth sensitivity was poorer for physically plausible than implausible objects. At first glance, our data are at odds with the findings of Wong et al. (2020). However, we view that the findings generated by both studies are in fact complementary to each other. The data from Wong et al. (2020) suggest a reweighing of stereo processes based on the physical plausibility of the object. They suggested that during the perception of familiar (physically plausible) objects, stereo signals are down-weighed while object-level processing is up-weighed, thus optimizing the interpretation and interaction with these familiar, naturally relevant objects. While Wong et al. (2020) showed a general modulation of disparity processes by the object’s context (physical plausibility), we argue that our data, on the other hand, suggest a more specific contextual effect on stereo processing, that depends on the biological relevance of the object. Here, all three kinds of stimulus used in our study, namely the upright face, the inverted face and the random shape, are physically plausible. If the stereo re-weighing mechanism as suggested by Wong et al. (2020) only cares about whether the stimulus is physically plausible or not, one should expect no sensitivity differences to be observed across our stimuli. On the other hand, if the re-weighing mechanism is based more broadly on familiarity, then, one should expect a down-weighing of stereo sensitivity for face, especially the upright face, compared with the inverted face and the random shapes. Our data in the SNR task are in line with this prediction. However, there is something more to unravel for the feature task, for which we observed a somewhat reversed behavioral trend. Here, we suggest that the everyday face perception tasks, such as face identification, recognition of facial expressions, etc., require the processing of precise details in the stimulus. Therefore, during the perception of faces, it would be likely that a pre-existing circuitry that favors detailed visual processing would be triggered, which in turns facilitates the readout of features. Critically, such facilitation in feature readout should be robust and able to counteract or even out-weigh the stereo down-weighing effect that results from the more general process as suggested by Wong et al. (2020).

### fMRI responses during depth judgments under varying object contexts

We next turn to our fMRI data. The GLM revealed rather homogenous univariate activation across stimulus conditions during depth judgments. Intriguingly, there was an overall higher amplitude of responses in the left versus right hemisphere. We speculate that this asymmetry has nothing to do with stereoscopic vision per se, as indeed, existing work has indicated an absence of hemispheric effects (Akay and Celebi, 2009) in depth perception. We fathom instead that our results might have been caused by the strong imbalance of genders in our recruited participants, with many more males than females in our fMRI component. To this end, there is evidence of globally stronger hemispheric asymmetry in males than in females (Hirnstein et al., 2019).

Our MVPA results were more revealing. We reasoned that we may be able to detect functionally-relevant responses to face-object modulations of stereo sensitivity, by teasing out any regions that can uniquely and commonly discriminate response patterns for the two stimulus contrasts involving the upright face stimulus (i.e., upright face vs random shape; upright face vs inverted face), but not for the comparison involving the inverted face and random shape. This allows us to avoid, as far as possible, picking up regions that might be responding to lower-order stimulus differences. We found that of our ROIs, only FFA showed unique above-baseline SVM accuracy for those classifications that involve the upright face, suggesting a potentially key role for this region for explaining the strong modulations of stereo-sensitivity by biological relevance that was observed behaviorally.

V3B, which was uniquely discriminative of upright face versus inverted face responses here, has been previously implicated in the discrimination of correlated and anticorrelated RDS (Preston et al., 2008). Both V3 (Backus et al., 2001; Anzai et al., 2011) and the inferotemporal cortex (Janssen et al., 2000, 2003) more broadly, have also been well demonstrated to be stereosensitive. Of these two regions, V3 showed here particular relevance to behavior (i.e., the upright vs inverted face, brain-behavior correlation). Here, larger discriminability of the two stimuli exhibited by V3 translated to larger discrepancies in the behavioral sensitivities to the two stimuli. While modulations in early visual cortex, long believed to care only about local stimulus features appear perplexing, this finding appears to well fit well fit previous work showing modulation of responses in V1 to ambiguous stereograms by changing perceived depth via luminance changes of the surround (Rideaux and Welchman, 2019).

Responses of retinotopic V1, V2, V3A, V7 found here likely reflect decoding of lower order structural differences between the objects during depth judgments as these regions showed above-baseline SVM accuracy in both classifications involving the random shape (upright face vs random shape and inverted face vs random shape). Further, above-baseline classification accuracies in the ventral regions (V4, LO) found in both comparisons likely reflect the decoding of the object’s identity and/or lower-order stereo information: V4 has been shown to respond to absolute depth (Shiozaki et al., 2012) and structured objects, regardless of the exact object identity (Desimone et al., 1985; Merigan and Pham, 1998). LO is well demonstrated to respond more strongly to intact than scrambled objects (Malach et al., 1995), and shows similar responses to familiar and novel shapes (Margalit et al., 2016). These findings are in line with our results where pattern discriminability was similar across classifications, although the upright and inverted faces differ in the level of familiarity.

We showed that depth sensitivity is affected by the biological relevance of the objects, defined here in terms of whether the target object depicted a face or non-face, and that such modulations are task-dependent. Using fMRI, we observed modulations of multivariate fMRI responses during depth judgments, particularly in FFA, by object type. We speculate that FFA is not only involved in object analysis as discussed above, but exerts considerable influence on stereoscopic mechanisms as early as in V3 such that the utility of disparity information is up/down regulated depending on the relevance of the object (i.e., whether it is a face or non-face). The role of FFA in face perception is well documented (Kanwisher et al., 1997, 1998), but its up-stream and down-stream interactions with more rudimentary processes (here, stereo-encoding, in particular carried by V3) is well worth continued exploration, perhaps through using more temporally-resolved techniques. Lastly, we fathom that our findings in the FFA likely reflect our choice of biological manipulations here (i.e., faces or non-faces). In this vein, it would be further interesting to explore object-stereo interactions under different indices that vary biological importance.

## Synthesis

Reviewing Editor: Morgan Barense, University of Toronto Decisions are customarily a result of the Reviewing Editor and the peer reviewers coming together and discussing their recommendations until a consensus is reached. When revisions are invited, a fact-based synthesis statement explaining their decision and outlining what is needed to prepare a revision will be listed below. The following reviewer(s) agreed to reveal their identity: Reuben Rideaux.

This paper assesses whether the biological relevance of an image defined by binocular disparity influences the way in which the binocular disparity is processed. This question is assessed using a combination of psychophysical and imaging (fMRI) methods. In the psychophysical experiment, the author/s compare the signal detection and feature difference thresholds for binocular disparity defined images of upright, inverted, and spatially scrambled faces. Findings indicate that the signal detection is better for inverted and scrambled faces, whereas discrimination of feature differences is performed better for upright and inverted faces. In the imaging experiment activity in areas V3, LO, and FFA uniquely distinguished between upright and scrambled faces, whereas activity in V1 and V3A can uniquely distinguish between inverted and scrambled faces. Activity in V2, V7, and V4 could distinguish between upright or inverted and scrambled faces. The paper also presents an inter-individual correlation between a behavioural measure of discriminability and decoding accuracy in FFA.

The experiments appear to be conducted thoroughly, the methods are comprehensively described, and the results will be of interest to readers. However, there are some issues that need be addressed before the manuscript is suitable for publication.

Major issues

There may be an issue with the stimulus and the task. In the signal detection (SNR) task, observers make a near far judgement relative to the surrounding RDS. For the upright and inverted face stimuli, there is a clear gradient of disparity from the edge to the centre, peaking at the nose. This means that on far trials, largest absolute disparities will be spatially proximal to the comparison surround, while on near trials the largest absolute disparity will be in the centre, far from the surround. Thus, one would expect that the task is easier for these stimuli on far trials. By contrast, no such asymmetry should exist for the scrambled face. The authors should test for this asymmetry and whether it influenced the pattern of performance between stimulus conditions on this task.

Related to the previous point, the results of the behavioural task were surprising, and given their unintuitive nature the authors should show the results of the staircases (which can be more susceptible to lapses than method of constant stimuli), and provide linked individual threshold datapoints across the three conditions (e.g., semi-transparent dots linked by lines overlaid on the bar plots).

We appreciate that the authors were careful to avoid directly comparing the results from the two MVPA analyses, however, by comparing the regions that were discriminable in one analysis but not the other and drawing inferences about the information encoded therein, it felt that this was implied. It would make sense to show the results of decoding upright from inverted faces. If this analysis yields no significant regions, this should be acknowledged, as currently there appears to be inferences drawn that are statistically unsound, i.e., if A and B are different but A and C are not then B and C must be different.

The behavioural-imaging correlation does not seem robust and it is not clear if it adds significantly to the reported findings. For example, the author/s combine data from the inverted and random conditions to compare against the upright condition, but the explanation provided for this does not feel sufficiently justified. Is the result the same if the data are not combined? If the correlation is only present when the data are combined, but the combination isn't justified a priori then it should be probably be ignored.

Minor issues

"For any instances where a single staircase could not yield reliable estimates..” Please report how many of these there were.

It sounds like the number of voxels included in the classification analysis was held constant at 50 for all ROIs, is this correct? Were different numbers of voxels used?

Show a plot of behavioural results from the scanner, next to those from outside the scanner, for comparison.

Provide discussion of possible explanation for lateral beta weight asymmetry.

Section 4.2. - “Responses of retinotopic V1, V2, V3A, V7 found here likely reflect decoding of lower order structural differences between the objects...”. In the paragraph prior to this statement, the authors discuss the finding that activity in FFA and V3 were uniquely decodable from the scrambled stimulus. There appears to be a suggestion that FFA and V3 process higher order stereoscopic features of the stimuli, while V1, V2, V3A, and V7 process lower order features. This distinction make sense for FFA, but it is hard to see this for V3, given that V3A and V7 are highly sensitive to first-order binocular disparity. The authors should offer a separate explanation for why activity from FFA and V3 are “uniquely” decodable.

The results from Rideaux & Welchman (2019) may be relevant to the study, they demonstrated contextual effects on stereopsis in V1.
