# Supplementary figures and images for: Modulations of Depth Responses in the Human Brain by Object Context: Does Biological Relevance Matter?
Source: eNeuro. 2021 Jul 15;8(4):ENEURO.0039-21.2021. doi: 10.1523/ENEURO.0039-21.2021 (PMC8287874; doi:10.1523/ENEURO.0039-21.2021)

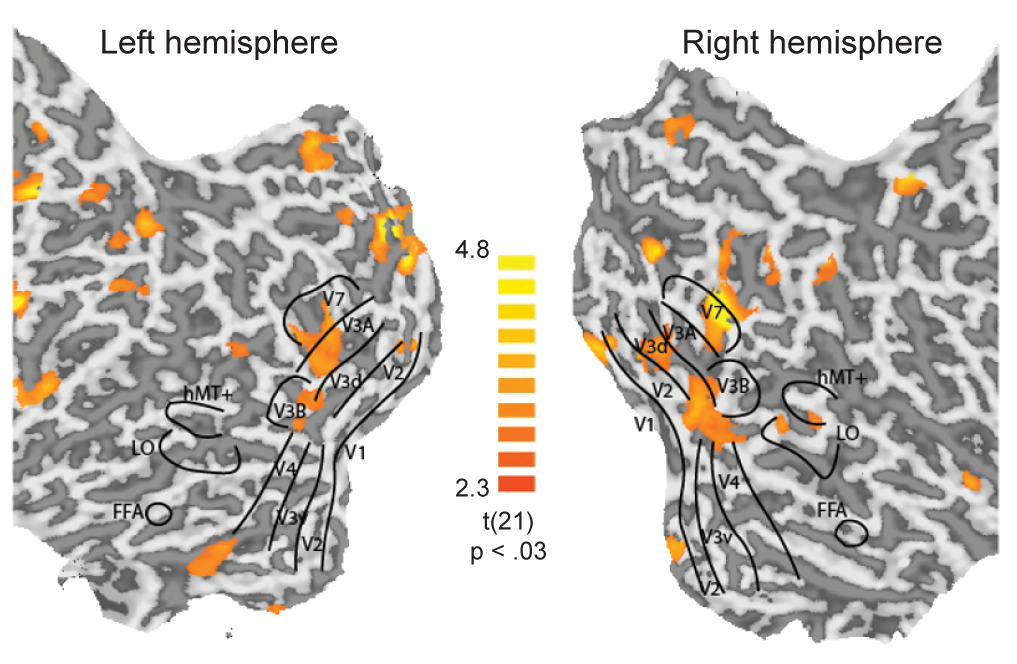

Supplement: Extended Data Figure 4-1 — Sample searchlight maps for SVM discriminations between the upright face and random shape conditions. Results are superimposed onto the representative flattened surface maps of a single participant with ROIs delineated. Gyri are colored in light grey and sulci in dark grey. Download Figure 4-1, TIF file. [file enu-eN-NWR-0039-21-s03.tif]

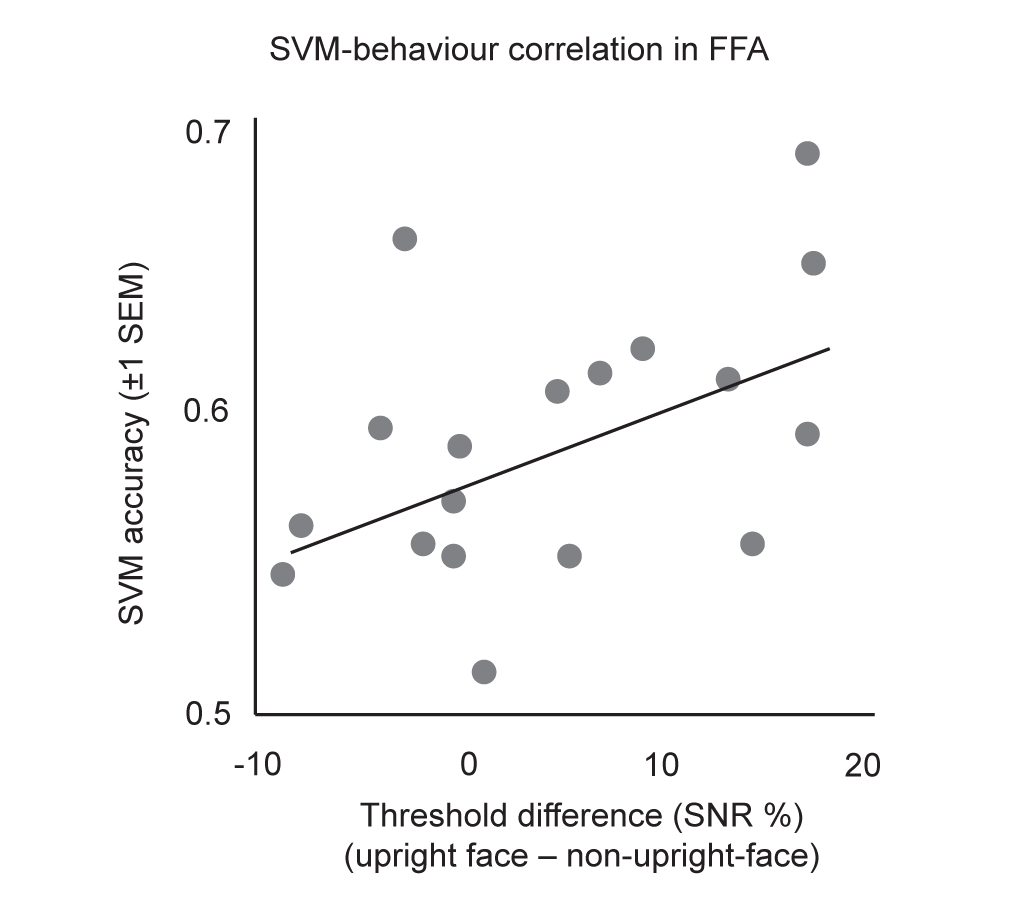

Supplement: Extended Data Figure 4-2 — Brain-behavior correlation in FFA for the upright-face versus non-face (i.e., concatenating the inverted face and random shape conditions) comparison. The horizontal axis represents a behavioral index computed as the threshold difference between the upright face condition and the mean of the two non-face conditions. Download Figure 4-2, TIF file. [file enu-eN-NWR-0039-21-s04.tif]
